# Identification of New Drug Targets and Resistance Mechanisms in *Mycobacterium tuberculosis*

**DOI:** 10.1371/journal.pone.0075245

**Published:** 2013-09-23

**Authors:** Thomas R. Ioerger, Theresa O’Malley, Reiling Liao, Kristine M. Guinn, Mark J. Hickey, Nilofar Mohaideen, Kenan C. Murphy, Helena I. M. Boshoff, Valerie Mizrahi, Eric J. Rubin, Christopher M. Sassetti, Clifton E. Barry, David R. Sherman, Tanya Parish, James C. Sacchettini

**Affiliations:** 1 Department of Computer Science and Engineering, Texas A&M University, College Station, Texas, United States of America; 2 Infectious Disease Research Institute, Seattle, Washington, United States of America; 3 Seattle Biomedical Research Institute, Seattle, Washington, United States of America; 4 Department of Immunology and Infectious Diseases, Harvard School of Public Health, Boston, Massachusetts, United States of America; 5 Department of Biochemistry and Biophysics, Texas A&M University, College Station, Texas, United States of America; 6 University of Massachusetts Medical School, Worcester, Massachusetts, United States of America; 7 Tuberculosis Research Section, Laboratory of Clinical Infectious Diseases, National Institute of Allergy and Infectious Diseases, Bethesda, Maryland, United States of America; 8 Institute of Infectious Disease and Molecular Medicine, University of Cape Town, Cape Town, South Africa; 9 Howard Hughes Medical Institute, Chevy Chase, Maryland, United States of America; Université de Montpellier 2, France

## Abstract

Identification of new drug targets is vital for the advancement of drug discovery against *Mycobacterium tuberculosis*, especially given the increase of resistance worldwide to first- and second-line drugs. Because traditional target-based screening has largely proven unsuccessful for antibiotic discovery, we have developed a scalable platform for target identification in *M. tuberculosis* that is based on whole-cell screening, coupled with whole-genome sequencing of resistant mutants and recombineering to confirm. The method yields targets paired with whole-cell active compounds, which can serve as novel scaffolds for drug development, molecular tools for validation, and/or as ligands for co-crystallization. It may also reveal other information about mechanisms of action, such as activation or efflux. Using this method, we identified resistance-linked genes for eight compounds with anti-tubercular activity. Four of the genes have previously been shown to be essential: AspS, aspartyl-tRNA synthetase, Pks13, a polyketide synthase involved in mycolic acid biosynthesis, MmpL3, a membrane transporter, and EccB3, a component of the ESX-3 type VII secretion system. AspS and Pks13 represent novel targets in protein translation and cell-wall biosynthesis. Both MmpL3 and EccB3 are involved in membrane transport. Pks13, AspS, and EccB3 represent novel candidates not targeted by existing TB drugs, and the availability of whole-cell active inhibitors greatly increases their potential for drug discovery.

## Introduction

Given the alarming rise of resistance to tuberculosis (TB) drugs worldwide, the identification of novel drug targets is critical for the future of TB control [1]. Many attempts to develop new drugs for infectious diseases have employed a target-based strategy, for example conducting high-throughput assays of large compound libraries for inhibition of a critical enzyme/protein. Recent analyses of large-scale target-based screening campaigns suggest that this strategy has not been productive; for example, in a retrospective study of over 70 campaigns conducted at GlaxoSmithKline, only five compounds have progressed into drug development despite multiple screens [2]. This lack of success was due to a number of factors, including lack of whole-cell activity (i.e. cell-wall permeability) for high-throughput screening leads. In contrast, whole-cell screening has numerous advantages, since compounds with demonstrable inhibition of bacterial growth can be directly identified from large compound libraries. This approach has the benefit that a genome-wide panel of essential cellular targets can be evaluated in a single assay; such a comprehensive approach avoids bias in target selection and obviates the need for detailed biological characterization of targets in advance. However, this approach is limited by the effort required to define the cellular targets of each compound, needed to facilitate subsequent medicinal chemistry.

We have developed a scalable platform for the discovery of drug targets for any pathogenic organism that is based on combining high-throughput screening (HTS) with whole-genome sequencing (WGS) of resistant isolates. The method is not biased by prior expectations of gene essentiality, and instead is driven by empirical observation of cellular processes whose inhibition leads to cell death. The approach begins with a whole-cell screen to identify compounds that inhibit growth. Bacterial mutants that are resistant to each active compound are selected *in vitro*, and deep sequencing is performed to identify resistance-associated polymorphisms. The contribution of each mutation to the resistant phenotype is then confirmed by introducing single point mutations into the parental strain by phage-recombinase-mediated recombination (i.e. “recombineering” [3]). Screening coupled with sequencing of resistant mutants has been used previously on an individual basis for identifying new targets, such as the F0-F1 ATP synthase, which was shown to be the target of diarylquinolines (e.g. Bedaquiline) at Johnson & Johnson [4,5]. The combination of next-generation sequencing and rapid selection and identification of resistance mutations we describe enables this method to be scaled-up effectively to allow for genome-wide surveys of novel inhibitor-target pairs.

Using this approach, each target is effectively pre-validated by demonstrating that the chemical inhibition of the protein leads to cell stasis or death. Furthermore, the inhibitory compound is by definition whole-cell active, making it a promising starting point for drug discovery (in that it can already be shown to gain cell entry). These compounds also provide a ligand for crystal structure determination of protein-ligand complexes. Additional information about the mechanism of action of a whole-cell active HTS hit can be uncovered through additional proteins implicated in resistance, such as activators, detoxification proteins, drug efflux pumps, or proteins affecting cell-wall permeability. The novelty of our approach is based on the scalability of the process; we have developed and refined methods for each technique which, in combination, provide a rapid, concurrent methodology for target identification that minimizes resource requirements and can be used for compounds for which there is limited supply.

In this study, resistance-associated mutations were identified for eight HTS hits with activity against *Mtb* using the described method, and the functional significance of the mutations has been confirmed via recombineering. The resistance-conferring mutations observed indicate a diverse range of possible resistance mechanisms. Four genes are essential for bacterial growth, and these mutations likely alter drug binding to its target site. Two genes are transcriptional regulators which might regulate expression of an efflux pump. One is a known pro-drug activator. Although most mutations consisted of single-nucleotide polymorphisms or small insertions/deletions, one compound had the unprecedented effect of selecting for the insertion of an IS*6110* transposon into a specific susceptibility-conferring gene. Our results in applying this target identification procedure to *Mtb* show that it is effective at discovering resistance-associated genes, including a subset of candidate drug targets.

## Results

**Figure 1:**
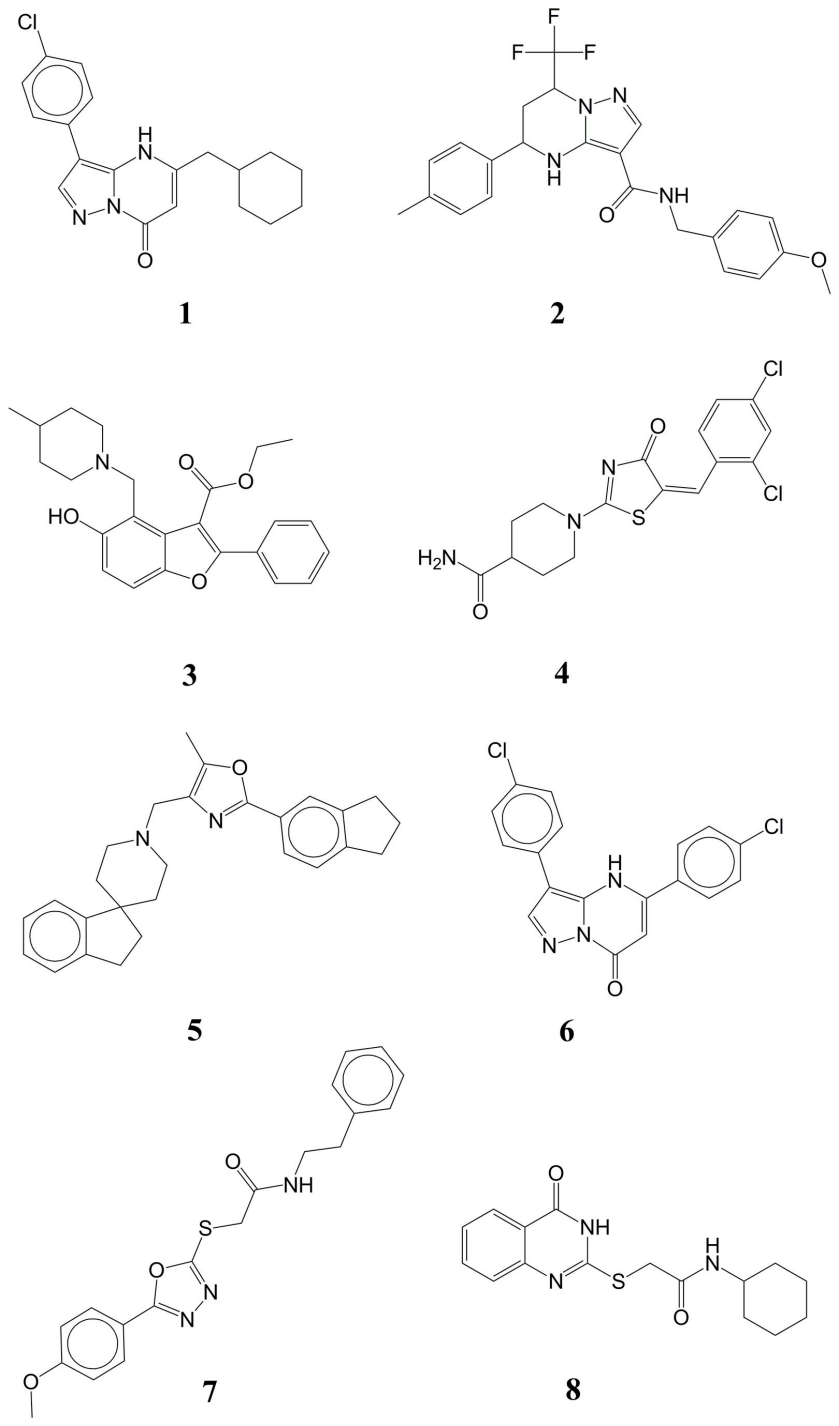
Compounds with whole-cell inhibition used for target identification.

To initiate this study, eight compounds with anti-tubercular activity (Figure 1) were selected from whole-cell screens against *Mtb* H37Rv performed at several institutions, including the National Institutes of Health, the University of Illinois at Chicago, and Novartis, Inc. High-throughput screening was carried out in liquid culture (7H9 medium) under aerobic conditions, using glucose as a carbon source, or in 7H12 medium with palmitate as carbon source. Whole-cell active compounds were selected from the screens using several criteria, including potency and chemical-structural properties. Compound activities were confirmed by evaluating the minimum inhibitory concentration (MIC_99_, representing 99% growth inhibition) in dose-response studies. MICs (on solid medium) ranged from 0.25 to 12.5 µM (Table 1). The selected compounds all have drug-like properties [6] in terms of molecular weight (<500) and logP (<6) (Table 1). Sources of compound material are listed in Table S1 in File S1.

**Table 1:** Molecular properties and MIC_99_ values of inhibitory compounds against *Mtb* H37Rv.

**compound**	**MW**	**logP**	**liquid MIC (μM**)	**solid MIC (μM**)
1	342	4.68	0.6	2.5
2	444	3.82	1.3	6.25*
3	395	4.15	2.0	6.25*
4	384	2.99	0.7	12.5*
5	399	5.71	0.6	0.25
6	356	4.26	12.5	5.0
7	369	2.65	>20	12.5
8	317	2.53	0.6	1.56*

^*^ These MIC_99_ values were determined in *Mtb* strain mc^2^ 7000, which is a Δ*panC*Δ*RD1* deletion mutant of H37Rv [70], but displays an identical phenotype and sensitivity to all drugs tested to date.

Resistant mutants of *Mtb* H37Rv were isolated for each compound on solid medium using methods optimized for *Mtb*. In order to obtain resistant isolates, we plated 10^7^, 10^8^ or 10^9^ cells on several concentrations of each compound above the MIC (2X, 5X, 10X solid MIC, or 5X and 20X liquid MIC). In order to conserve compound, which in some cases was limiting, we used a minimal quantity of agar (3 ml) in 12-well plates. The combination of compound concentration and CFUs (colony-forming units) which gave rise to individual resistant colonies was compound-dependent. For each compound we performed the isolation protocols 1-3 times. Colonies were obtained for all compounds. For each compound, we selected three to four resistant isolates for whole-genome sequencing. This allowed the comparison of observed genetic polymorphisms among independent clones. For a subset of the compounds, the MIC_99_ of resistant colonies was determined by serial dilutions of the compound on solid media (plates). For the remaining compounds, because the amount of material was limited, a lower bound for the MIC was determined in liquid media at several concentrations above the wt MIC and reported as the highest concentration at which growth was observed (the MIC could have been even higher than the highest concentration tested). In each case, resistant mutants had a shift of at least 4-fold over the relevant wt MIC, except for compound **1** (Table 2).

**Table 2:** Selection of resistant mutants.

**compound**	**concentr. used for selection (μM**)	**multiple over MIC (solid media**)	**MIC of mutants (μM**)	**method used to determine MIC**	**relevant reference MIC (μM**)** (from Table 1**)	**multiple over wt MIC**
1	12.5	5x	6.3	solid	2.5	2.5x
2	26	4x	≥10	liquid	1.3	≥7.5x
3	10	1.6x	≥16	liquid	2.0	≥8x
4	12	1x	≥32	liquid	0.7	≥46x
5	1.25	5x	2.5	solid	0.25	10x
6	25	5x	50-100	liquid	12.5	≥4x
7	50	4x	>100	solid	12.5	>8x
8	3, 12	2x,10x	≥10	liquid	0.6	≥16x

For some of the mutants, the MIC_99_ was determined by serial dilution on solid medium. For the other mutants, the MIC growth was assessed in liquid medium at several concentrations above the wt MIC, and the highest concentration at which growth was observed is reported, thus representing a lower bound for the MIC (marked as '≥').

Genomic DNA for resistant clones was extracted and sequenced using an Illumina GenomeAnalyzer IIx (GAIIx). The samples were sequenced in paired-end mode with a read length of 36 to 51 bp (with pairs of reads from opposite ends of ~200 bp genomic fragments). Sequencing details for the individual strains are shown in Table S2 in File S1. Reads were assembled into complete genome sequences using a comparative assembly method [7]. The genomes of the parental strains used (H37Rv stocks from each individual laboratory) were first sequenced, and these were used as reference sequences for assembly, to screen out polymorphisms that are common to the parental strain used. The depth of coverage varied depending on the sample, but ranged between 21 and 174 (i.e. mean number of reads covering each site, averaged over the whole genome). The completion for each genome was >97.8% (percentage of 4,411,532 sites covered by at least 2 reads), and regions not covered were primarily restricted to PE_PGRS genes, which often have low coverage due to high GC content and generally are not relevant to drug resistance in TB [8]. The mutations identified in each strain are shown in Table S2 in File S1. The table includes all polymorphisms that could be identified with high confidence (where coverage 5X and homogeneity of base call >70%).

Table 3 summarizes the mutations found in common loci for each compound. For each compound, mutations could be identified either at an identical site or within the same gene among all the resistant mutants. No common mutations were found in strains resistant to different compounds. In most cases, each resistant mutant for a given compound had a non-synonymous substitution in the same gene. In some cases these mutations altered different codons within the same gene, or occurred in the upstream non-coding region (e.g. putative promoter mutations -44 bp upstream of *ndhA*). No large-scale insertions, deletions, or duplications were detected among these resistant mutants. Insertion locations of the IS*6110* transposon were identified, and were found to be identical to the 16 sites in H37Rv in all cases except one (Rv1685c, *vide infra*).

**Table 3:** Polymorphisms identified in resistant mutants.

**compound**	**ORF**	**gene**	**relevant polymorphisms observed**	**# mutants explained/ sequenced**	**essential in vitro?**	**function**
1	Rv0283	*eccB3*	R14L, N24H, -AAC in aa 26	3/3	Yes	component of ESX-3 type VII secretion system
2	Rv0206c	*mmpL3*	F644L (2), F644C, A677V	4/4	Yes	membrane transporter
3	Rv3800c	*pks13*	D1644G,D1607N (3)	4/4	Yes	polyketide synthase (mycolic acid biosynthesis)
4	Rv2572c	*aspS*	F526L,T570I (2)	3/3	Yes	aspartyl-tRNA synthetase
5	Rv0678		I67S, aa69 +GC, +A at -9 promoter	3/3	No	transcriptional regulator of *mmpL5*/*mmpS5* efflux pump
6	Rv1685c		IS6110 transposon insertions in aa 96, 101, and 105	3/3	No	transcription factor
7	Rv3854c	*ethA*	C253R, -T in Q24 , -T in E113	3/3	No	monooxygenase (ethionamide activator)
8	Rv0392c	*ndhA*	G>C -44 bp upstream	4/4	No	NADH dehydrogenase

Essentiality was determined *in vitro* via transposon mutagenesis analyzed via deep sequencing [9]. These are consistent with earlier analyses of essentiality based on PCR sequencing of transposon mutants [71].

The essentiality of these genes *in vitro* was previously determined by transposon mutagenesis studies using a high-resolution method based on deep-sequencing that yields data on insertions at individual TA dinucleotide sites [9]. Four of the resistance genes identified are known to be essential, suggesting they are direct targets of the respective inhibitors. The causes of resistance to each compound are discussed on an individual basis below.

Recombineering was used to validate the functional significance of the resistance-associated polymorphisms (Table 4). Mutations identified in resistant mutants were transferred to the parental strain using the phage Che9c recombination functions [10], which was optimized for efficient integration of oligonucleotides into the chromosome of *Mtb*. The protocol is designed to select specifically for resistant transformants while minimizing the risk of selecting oligo-independent resistant mutants. Using the recombineering plasmid pKM402, which expresses only the RecT function (the only one required) at high levels from the Ptet promoter, the rate of oligo-mediated recombineering is 4 orders of magnitude higher than the spontaneous rate of mutation, greatly increasing the confidence in identification of oligo-mediated changes for SNP verification. In seven of the eight cases, transformants with the predicted mutations were shown to tolerate higher inhibitor concentrations than wild-type (producing colonies on plates at concentrations at which none or a much lower frequency were observed for no-DNA controls), confirming that the identified mutations are responsible for conferring resistance.

**Table 4:** Recombineering results.

**compound**	**solid MIC (μM**)	**concentration (μM**)** at which colonies of transformants were observed, but not for no-DNA controls**	**multiple over solid MIC**	**mutation recombineered**	**colonies observed?**
1	3.1			*eccB3:* R14L, N24H, -AAC in aa 26	no
2	6.25	26	5x	*mmpL3:* F644L,F644C,A677V	yes
3	6.25	10	1.6x	*pks13:* D1644G, D1607N	yes
4	12.5	32	2.5x	*aspS:F526L*	yes
5	0.25	1.0	4x	Rv0678: I67S, aa69 +GC, +A at -9 promoter	yes
6	5.0	15	3x	Rv1685c: frameshifts at same sites as IS6110 insertions	yes
7	12.5	100	8x	*ethA:* C253R, -T in Q24	yes
8	1.56	12	8x	*ndhA:g-44c*	yes

### EccB3

Clones selected for resistance to **1** were found to have MICs 2.5-fold higher than the parental strain (on solid medium). Of the 3 resistant mutants sequenced, all three had mutations in *eccB3* (Rv0283), including two that caused non-synonymous amino acid substitutions (R14L, N24H), and one that introduced a frame-preserving deletion of three nucleotides (-AAC, deleting amino acid 26). EccB3 is a component of the ESX-3 type VII secretion system, one of five paralogous clusters (ESX-1 through ESX-5) in the *Mtb* genome [11]. Among the five ESX clusters in the TB genome, only ESX-3 is essential *in vitro* [9]. ESX-3 had been found to be regulated by the FurB transcription factor, suggesting that it is involved in iron or zinc acquisition [12]. Two lines of evidence strongly suggest that this system is absolutely required for iron and zinc acquisition. Strains of *Mtb* [13] and *Mycobacterium bovis* BCG [14] in which the native promoters for this locus were replaced by artificially regulated promoters were unable to grow without excess iron and zinc. Moreover, a *Mycobacterium smegmatis* strain lacking the entire ESX-3 locus and a second iron uptake system also could not grow in iron-depleted medium [14]. Loss of ESX-3 was specifically associated with the inability to utilize a mycobacterial siderophore, mycobactin [14]. Intriguingly, compound **1** has a pyrazolo(1,5α) pyrimidinone core which could potentially chelate metal ions or participate in redox cycling. The precise role of the EccB3 subunit in the ESX-3 secretion complex is unknown. All three mutated residues are localized in the predicted 73 amino acid cytosolic domain on the N-terminal side of this protein’s single predicted transmembrane region. However, recombineering of these mutations did not yield transformants that were resistant to **1**. The reason for this is unclear, but it might be that special conditions such as selection on iron-depleted media are required to successfully obtain resistant transformants.

### MmpL3

**Figure 2:**
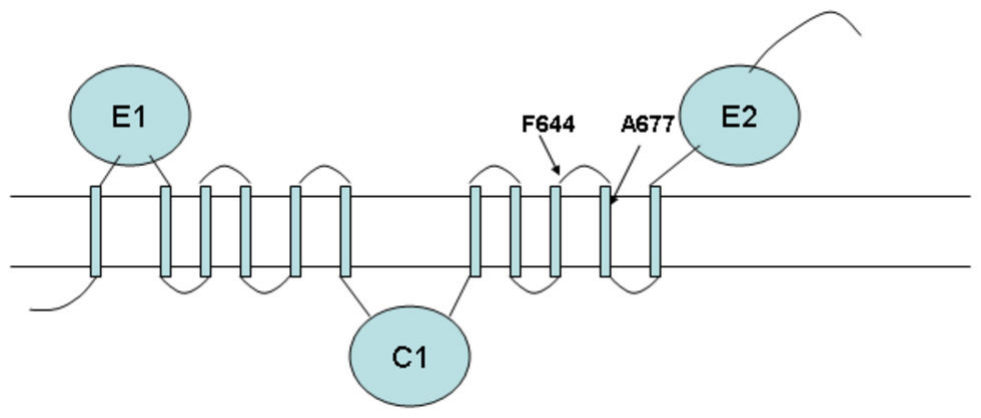
Location of mutations observed in MmpL3 (based on Tullius et al., 2011).

Four mutants resistant to **2** were found to have mutations in *mmpL3* (Rv0206c), translating to F644L (2), F644C, and A677C. These mutants had MICs of at least 10 µM (liquid), or 7.5X the MIC of the parental strain (1.3 µM, liquid). MmpL3 is a membrane transporter in the RND (Resistance-Nodulation-Cell Division) family, and is predicted by transposon mutagenesis [9] as well as targeted inactivation by recombination [15] to be the only essential member of the eleven MmpL gene family of *Mtb*. MmpL3 has recently been shown to be the target of several small molecules with diverse chemical structures, including BM212 [16], SQ109 [17] and AU1234 [18]. MmpL3 is predicted to have two extracellular domains and a central cytosolic domain, each separated by multiple membrane-spanning alpha-helices (Figure 2). The biological role of MmpL3 in the cell is currently unclear. It has been implicated in transport of iron [19], mycolic acids [18], and trehalose mono-mycolate [17]. One of the resistance mutations for **2** (A677V) is located in a membrane-spanning helix between the cytosolic and C-terminal extracellular domains (see Figure 2), and the other mutation is in a nearby helix-connecting loop (F644L). Most of the mutations associated with resistance to BM212 are conservative (hydrophobic) and are also observed to fall in the transmembrane helices (including A254V, I296L, I249T, F240L, L196P, A347V, V689G, in the *M. smegmatis* ortholog, MSMEG_0250) [16]. It is possible that these mutations (for both BM212 and **2**) occur in the inner annulus of a pore formed by the 11 transmembrane helices, and thus influence how these inhibitory compounds interfere with uptake or efflux of an essential metabolite. Alternatively, MmpL3 might be acting as an efflux pump to expel these compounds from the cell and the observed mutations might enhance affinity. However, it is noteworthy that the bactericidal activity of BM212 (1.5 µg/ml MIC for H37Rv) was not influenced by treatment with efflux pump inhibitors reserpine and verapamil [16], suggesting that MmpL3 may represent the direct target of this compound, and that the associated mutations in this gene are not likely to confer resistance through increased efflux. When the mutations observed in *mmpL3* were independently transferred to the parental strain via recombineering, colonies of transformants were observed on plates containing **2** at a concentration of 26 µM (5X the MIC of the parental strain on solid medium), confirming a causal link to resistance.

### Pks13

**Figure 3:**
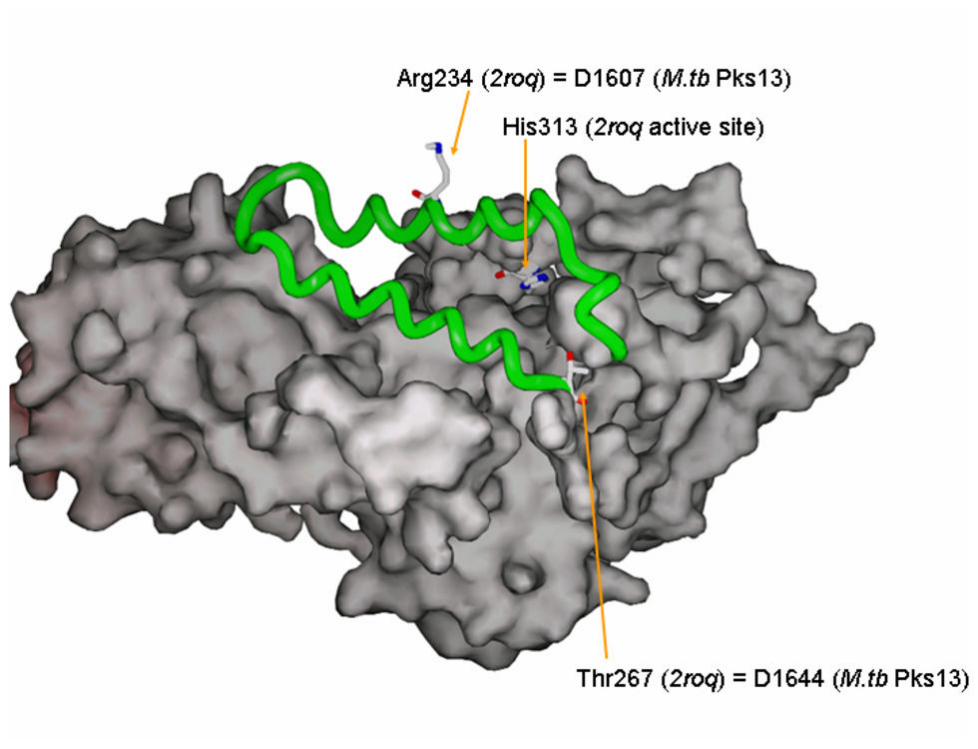
Location of mutations in *Mtb* Pks13, mapped onto the thiolation-thioesterase domain of *E. coli* enterobactin synthase (EntF, PDB: 2roq). The mutations occur in a 2-helix lid that closes over the active site.

Compound **3**, a benzofuran with an MIC in liquid medium of 2.0 µM, selects for mutations in *pks13* (Rv3800c), including D1607N and D1644G. Resistant mutants had >8-fold higher MICs than the parental strain (evaluated in liquid medium). Pks13 is known to be essential for growth *in vitro* [20,21] and is a multi-domain polyketide synthase involved in mycolic acid biosynthesis [22]. The enzyme catalyses condensation of a C_24_-C_26_ long-chain fatty acid to a C_40_-C_60_ meromycolate chain, forming the α-alkyl-β-ketoester branched-chain precursors of mycolic acids. The enzyme consists of 5 functional modules: a keto synthase domain, acyltransferase domain, 2 acyl-carrier protein (ACP) domains, and a thioesterase domain. Pks13 has been shown to physically interact with FadD32 [22], a fatty acyl-AMP ligase that is also essential [20], which primes the acyl substrate for transfer onto one of the ACP domains of Pks13 via adenylation. The mutations observed in mutants resistant to compound **3** (D1644G and D1607N) were located in the C-terminal thioesterase domain (residues 1400-1700), suggesting that the inhibitor could block de-esterification of the product (branched mycolic acid precursor). Homology modeling based on the thiolation-thioesterase (di) domain of *E. coli* enterobactin synthase [23] (EntF, PDB: 2roq), the closest homolog for which a structure is known (25% amino acid identity), shows these mutations are located in a loop consisting of a pair of alpha-helices that cover the active site and form a lid that must open up to accept the pantothenate moiety of the substrate (Figure 3). Hence the mutations could affect the lid structure in such a way as to prevent inhibitor binding in the active site while maintaining native thioesterase function. Using recombineering to transfer the mutations into a clean genetic background, colonies were produced on plates containing **3** at 10µM, a concentration at which no colonies in the wild-type control were observed, confirming their link with resistance

### AspS

**Figure 4:**
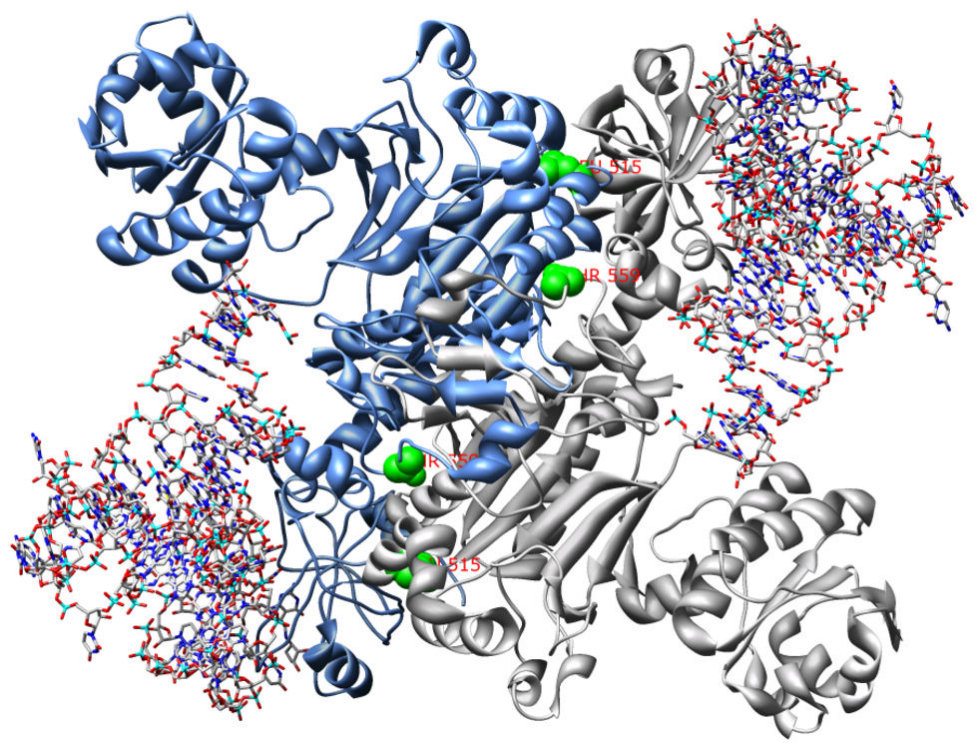
Location of mutations in AspS, based on the *T. thermophilus* model (PDB: 1efw). The peptide chains are shown as blue and gray ribbons, and the tRNA molecules are shown as atomic models. The side-chains of residues Leu515 and Thr559, corresponding to the mutations in the Mtb AspS (F526 and T570), are highlighted in green and are located in the dimer interface.

All three mutants resistant to compound **4** harbored mutations in *aspS* (Rv2572c), a class II aspartyl-tRNA synthetase. Compound **4** has a 4-thiazolidinone core, and other compounds with this core have been found to have antitubercular activity [24]. Mutants resistant to **4** had MICs in liquid medium of 32 µM, which is 46-fold higher than the MIC of the parental strain, 0.7 µM. AspS is predicted to be essential [9], presumably because of its critical role in protein synthesis, and the lack of an alternative aspartyl tRNA synthetase in the *Mtb* genome. The mutations observed in the resistant strains sequenced were F526L and T570I (2). Interestingly, these residues occur on the surface of the protein, not in the amino-acylation active site. Based on the crystal structure of the *T. thermophilus* AspS enzyme (1EFW [25]), these residues (corresponding to residues L515 and T559) both appear near the dimer interface, approximately 15 Å apart on opposite subunits (Figure 4). Thus, it is possible that inhibitor binding to this region disrupts dimerization, which is necessary for the activity of the yeast homolog [26]. Transfer of the F526L mutation in *aspS* to the parental strain via recombineering allowed colonies to grow on plates with compound **4** at 32 µM (absence of colonies in the wild-type control well at this concentration indicated resistance).

### Rv0678

Selection for resistance to **5** on plates at a concentration of 5X the solid MIC of H37Rv resulted in mutations in *rv0678*, a transcriptional regulator. Three distinct mutations were observed: a non-synonymous SNP (I67S), a frame-shifting insertion +GC in residue 69, and an insertion of +A at -9 bp upstream of the coding region (possibly in the promoter or ribosome binding site). Resistant mutants had a 10-fold increase in MIC (2.5 µM) compared to wt (0.25 µM). The two mutations in the coding region are likely to cause loss-of-function, and we hypothesize that the upstream mutation would lead to a decrease in expression. Since Rv0678 is a negative regulator of MmpL5 and MmpS5 [27], these mutations in Rv0678 may lead to the increased expression of this putative transporter. qRT-PCR expression studies of the resistant mutant to **5** with mutation I67S indicate significant up-regulation of both Rv0676 (MmpL5) and Rv0677 (MmpS5) (Table S3 in File S1). Similar mutations in *rv0678* (including the +A promoter mutation at -9 bp upstream, as well as other frameshifts) result in resistance to certain azole-containing compounds (antifungals), which is proposed to be due to increased efflux of the compound [27]. **5** contains an oxazole moiety, and thus drug efflux by the MmpL5-MmpS5 system is a plausible mechanism by which mutations in *rv0678* confer resistance. We checked for cross-resistance of these strains to azoles, and found that one mutant had a slight increase in resistance to econazole (25 µM MIC, 2.5-fold higher than for H37Rv), though the other two did not. All three mutations were recombineered and showed growth in wells with 4X the solid MIC, a concentration at which no growth was observed for the parental strain.

### Rv1685c

Mutants were selected for resistance to **6** on plates with a concentration 5X the parental MIC (solid), and all three mutants sequenced exhibited insertions of an IS*6110* insertion sequence (transposon) in *rv1685c*. Rv1685c is a transcription factor whose biological role and regulatory targets are not known, though it does not appear to be essential *in vitro* [9]. Rv1685c resides in an operon and is co-expressed with components of a predicted ABC transporter (Rv1686c, Rv1687c). Thus resistance to **6** could be due to efflux. The insertions of the IS*6110* transposon were located at distinct but proximal sites in each of the 3 resistant mutants sequenced: in amino acids 96, 101, and 105 (in the middle of the 207-residue protein product). These insertions presumably disrupt the function of the gene product. These mutants had MICs >4X the parental MIC (liquid). Since insertion of IS6110 sequences in target locations is technically challenging, we instead recombineered both frame-shift and stop-codon mutations at the same sites where the transposon insertions were observed in the resistant mutants. In each case, transformants were observed at concentrations 3 times higher than the solid MIC of wt H37Rv, confirming that disruption of Rv1685c confers resistance to **6**.

Note that compounds **1** and **6** display a high degree of chemical similarity, with a purine-like core (pyrazolo(1,5α) pyrimidinone); yet they clearly select for polymorphisms in distinct genes. It is possible that they ultimately inhibit the same target, such as ESX-3, although resistance to compound **6** might be achieved by a distinct mechanism, e.g. efflux.

### EthA

Resistant mutants to **7** (an oxadiazole thioether) displayed greater than an >8-fold shift in MIC (>100 µM) on solid medium. Similar oxadiazole thioesters have been shown to have antifungal activity [28]. Three clones were selected for sequencing. One mutant resistant to **7** was sequenced and found to have a non-synonymous polymorphism in *ethA* (Rv3854c), corresponding to amino acid substitution C253R. The other two had frame-shifts: -A in codon 24 and -A in codon 113 in *ethA*. EthA is a non-essential FAD-dependent monooxygenase that is a known activator of the second-line anti-tuberculars ethionamide, prothionamide and thiacetazone [29,30,31,32]. Thus, EthA could also activate **7** which contains a thioether-substituted oxadiazole and may hydrolyze to form an activated thiocarbonyl that could be oxidatively activated by EthA similar to ethionamide. However, the C253R mutant was not found to be cross-resistant to ethionamide. The C253R substitution and one of the indels (-A in codon 24) were recombineered, and both showed growth of transformants in wells at 8X the solid MIC, whereas the untransformed parental strain did not. Selection and sequencing of additional mutants and biochemical characterization will be needed to identify the ultimate target of **7**.

### NdhA

Mutants resistant to **8**, a 2-mercapto-quinazolinone, were selected on plates with 2X or 10X the solid MIC of the parent. Four resistant mutants were sequenced, and all four contained a single nucleotide substitution of G to C located 44 bp upstream of the coding region of *ndhA* (Rv0392c). Mutants with the *ndhA*:C-44G mutation had liquid MICs of 10 µM (16X the parental MIC of 0.6 µM). NdhA is a membrane-bound type-II NADH dehydrogenase which participates in energy metabolism, regenerating NAD^+^ via the oxidative phosphorylation pathway. However, NdhA is found to be non-essential *in vitro* and *in vivo* by transposon mutatgenesis [9,33], possibly due to redundancy with Ndh, the other type II NADH dehydrogenase in the *Mtb* genome (Rv1854c), which is essential [34]. One mechanism by which this mutation could confer resistance would be through up-regulation of NdhA expression, which could cause resistance by binding and sequestering the compound. Alternatively, it is possible that **8** acts by inhibiting an NADH-dependent target, and the up-regulation of NdhA might compensate for this by restoring the intracellular NADH:NAD^+^ ratio, analogous to a resistance mechanism for isoniazid [35]. Indeed, we evaluated the expression level of *ndhA* in the resistant mutants by qRT-PCR and found that expression was increased 40- to 80-fold over H37Rv, while 4 other transcripts (*ahpC*, *sigA*, 16S rRNA and 23S rRNA) were unchanged, confirming the hypothesized effect of this mutation in the upstream regulatory region. It is also possible the true target might be involved in nucleotide salvage or synthesis, as other molecules with a mercapto-quinazolinone core, have been found to bind and inhibit human purine nucleoside phosphorylase [36]. The *ndhA* C-44G mutation was introduced into the parental strain by recombineering, and was found to increase the resistance to **8**, yielding colonies of transformants when plated on concentrations up to 12 µM, implying an approximate 8-fold shift in MIC over the solid MIC of 1.56 µM for the parental strain.

## Discussion

The eight genes identified through this combined HTS/WGS method represent a range of resistance mechanisms. Based on essentiality analysis, four genes likely represent direct drug targets (Pks13, EccB3, AspS, MmpL3). Two are transcription factors that presumably regulate the expression of another protein that determines drug susceptibility, and one (EthA) is a known pro-drug activator, which could plausibly act by modifying the compound into a toxic metabolite. One of the targets is implicated in cell-wall biosynthesis (Pks13, synthesis of mycolic acids). This is not surprising, given the importance of this pathway (particularly fatty-acid biosynthesis) to the mycobacterium, and the fact that many existing drugs target this pathway, including isoniazid and triclosan (which inhibit InhA [37,38]). Recently, discovery of another inhibitor of Mtb Pks13 was reported [39], although resistance mutations for that compound (which is based on a thiophene core) appear in the N-terminal domain and it appears to inhibit the fatty-acyl-AMP loading of Pks13. This differs from the probable mechanism of action of compound **3** in this study, which likely interferes with the thioesterase activity in the C-terminal domain. Another target identified (AspS) is involved in protein translation, which is a common target for many antibiotics. There is precedent for tRNA synthetase inhibitors as antibiotics, such as mupirocin, a natural product that is thought to target the Ile tRNA synthetase in Gram positive bacteria [40], and inhibitors of methionine-tRNA synthetase that are being developed for treatment of MRSA infections [41]. The discovery of disruptions of *rv1685c* by IS*6110* transposition is a novel mechanism of resistance that is rarely observed in the laboratory in response to selection against inhibitors. IS*6110* transpositions have not, to our knowledge, been previously reported as a mechanism of drug resistance in the laboratory. However, IS*6110* transpositions can be induced synthetically under certain stress conditions, such as microaerophilic environments [42]. Furthermore, IS*6110* insertions in *rv1685c* have not been previously reported in clinical isolates of *Mtb* in association with drug resistance, although variation in IS*6110* insertion locations is observed among clinical isolates and is frequently used for epidemiological typing [43]. [42] IS*6110* transposition events were not observed in any of the other mutants we sequenced.

Resistance to at least two of the compounds is likely mediated though efflux. We observed no mutations directly in known drug efflux pumps, though resistance to the two compounds that selected for mutations in transcription factors (Rv0678 and Rv1685c, for compounds **5** and **6** respectively) is probably achieved through changes in regulation of chromosomally-adjacent membrane transporters (as was shown to be the case for Rv0678). Selection of resistant mutants in other organisms like *Pseudomonas aeruginosa* often leads to up-regulation of drug efflux pumps [44]. This would be a potential concern for the target identification approach we describe if mutations in targets were consistently masked by mutations in drug pumps. However, although *Mtb* has a large complement of membrane transporters that could act as efflux pumps (e.g. 13 RND resistance/nodulation/division proteins [15]; >30 ABC transporters [45]; and 16 in the MFS major facilitator superfamily [46]), induction of efflux pump expression does not seem to be the primary mode of resistance in *Mtb* [47,48]. Some drug efflux pumps in *Mtb* have been shown to have specificity for known drugs, including isoniazid (IniABC [49]), tetracycline (Tap, Rv1258 [50]), rifampicin (P55, Rv1410c [51]), spectinomycin (Stp, Rv2333c [52]) and fluoroquinolones (Rv2686c-Rv2688c [53]), though mutations in these genes or their regulators are typically not observed in drug-resistant clinical isolates or laboratory-derived mutants. In order to find the true intracellular targets of compounds like 5 and 6, it might be necessary to employ efflux-pump inhibitors such as reserpine or verapamil [54], or to use deletion mutants (e.g. of pump regulators) during mutant selection to suppress the occurrence of mutations that act through up-regulation of the pump, and thus force mutations in other genes to achieve resistance to the inhibitor. In the case of compound **6**, we noted that, despite the high degree of chemical similarity to compound **1**, they selected for mutations in different genes. It is possible that they both act in a similar manner (e.g. somewhere in iron metabolism as we suspect for **1**), though there might be multiple mechanisms for overcoming toxicity, potentially including efflux for **6**.

The approach we have described is an unbiased method for target identification that does not depend on prior expectations of which genes or pathways might be essential to an organism. Target-based approaches to drug discovery have been found to be effective in only a handful of cases (for example, peptide deformylase inhibitors [55]), but most other target-based efforts to date have failed, necessitating novel genome-wide methods for discovering new targets. Despite the fact that the *Mtb* H37Rv genome was sequenced over a decade ago, nearly half of the open reading frames remain annotated as hypothetical proteins whose functions are unknown, or are in highly duplicated families (e.g. FadD) where the specific functions and substrates remain to be elucidated. Intense effort has been invested in identifying “persistence” targets, or genes that are specifically essential for maintaining infection, as high-value targets for drug discovery [56]. The hallmark of TB infection is the latent (possibly non-replicating) state that a sub-population of bacilli appear to enter, in which they become more drug tolerant [57]. Genes implicated in maintaining this state, ranging from isocitrate lyase (a member of the glyoxylate shunt [58]) to DosR (regulator of the hypoxic response [59,60]) to MbtI (mycobactin biosynthesis, iron acquisition [61,62]), have all been investigated as drug targets, but these efforts have so far failed to yield effective lead compounds. An approach based on high-throughput screening starts with inhibitory compounds and works backwards to determine which proteins are revealed as vulnerabilities. This approach should greatly amplify the value of the large volume of high-throughput screening data for *Mtb* that has recently been made publicly available [63,64,65], by finding targets for these compounds. The optimized techniques and pipeline we have developed demonstrate that this method can be effectively scaled-up to identify a broad range of new targets as part of an intensive, full-scale drug discovery campaign against a pathogen.

## Experimental Procedures

### High-Throughput Screening

Compounds with whole-cell activity were selected from high-throughput screens run at several institutions, including the NIH, the University of Illinois at Chicago, and Novartis, Inc. All screens used similar conditions. *Mtb* H37Rv was grown at 37°C in 7H9 medium with glucose as a carbon source, or in 7H12 with palmitate as carbon source. After 2-3 weeks, cultures were tested for viability using the Microplate Alamar Blue Assay [66]. Compounds were evaluated at a concentration of 10 µM. Compounds demonstrating >50% inhibition were re-tested in dose response studies, and an MIC_99_ (99% growth inhibition) in liquid medium was determined.

### Selection of Resistant Mutants

Pilot experiments indicated that liquid MIC values did not necessarily translate directly to work on solid medium. Therefore, prior to selection, we determined the solid MIC_99_ for H37Rv wild type as described [67]. Resistant mutants were isolated by plating approximately 10^7^, 10^8^, and 10^9^ bacteria (in 100 µl volume) onto 12-well plates containing 2X, 5X and 10X solid MIC_99_ of compound, or 5X and 20X the liquid MIC. Resistant colonies were then re-streaked onto 2X MIC-containing medium, liquid cultures prepared, and solid MICs determined alongside wild-type H37Rv. Resistant mutants were typically isolated within 1-3 attempts of mutant selection using up to 10^9^ cells. The MIC_99_ of the selected colonies resistant to each compound was determined by serial dilution on solid media (plates). In cases where compound material was limiting, resistance was evaluated in liquid media at several concentrations above the wt MIC, reporting the highest concentration at which growth was observed. This placed a lower bound on the MIC (the MIC could have been even higher than the highest concentration tested).

### Genome Sequencing and Identification of Polymorphisms

Genomic DNA from resistant mutants was extracted by using either the method described in [68] or with ArchivePure DNA reagents from 5Prime.com. The DNA library was constructed by using a genomic DNA sample preparation kit (Illumina). The sample was first fragmented by either nebulization (provided in Illumina kit) or sonication (Covaris, Inc). The double-stranded DNA fragments comprised of 3′ or 5′ overhangs were converted into blunt ends, using T4 DNA polymerase and Klenow enzyme. Then Klenow Exo^-^ (lacking 3′-to-5′ exonuclease activity) was used to extend blunt ends with an “A” base, so that the fragments could be ligated to the adaptors (Illumina TruSeq kit, or NuGen), which have a single 5’ “T” base overhang. The ligated DNA was size selected on a 2% agarose gel. DNA fragments of 250-350 bp were excised from the preparative portion of the gel. DNA was then recovered by using a Qiagen gel extraction kit and PCR amplified to produce the final DNA library. Samples were typically multiplexed at 6-12 per lane, and a total of 5 pmol of DNA was loaded onto each lane of the sequencing chip for cluster generation. φX174 DNA was used as a control.

The sequencer was operated in paired-end mode, collecting pairs of reads from opposite ends of each fragment. The sequencing reaction was run for 36-51 cycles (read length in bp), and images of each tile on the chip were taken in different wavelengths for exciting each base-specific fluorophore. Image analysis and base-calling were done by using the Illumina GA Pipeline software (v0.3), or Off-Line Basecaller (OLB) v1.8. An additional read of length 4-7 bp was collected to determine the nucleotide bar code from the adapter ligation, and these sequences were used to de-multiplex the reads in each lane (assign to individual samples).

The reads that were generated for each strain were mapped against the appropriate reference genome sequence (aligned, allowing up to 2 mismatches and no gaps), based on the parental strain from which the mutants were selected (laboratory-specific variants of H37Rv that we have previously sequenced [7]). Paired-end constraints were applied by requiring both reads of a fragment to map to within 500 bp of each other. Apparent differences (i.e. sites where the consensus base from overlapping reads differed from the expected base in the reference sequence), along with sites where coverage was low or observed bases were heterogeneous, were identified, and a contig-building algorithm was used to construct a local ~200 bp sequence spanning the site to resolve the sites into SNPs versus indels, based on alignment to the corresponding region in the reference genome. We systematically searched for evidence of large-scale deletions (regions >100 bp with <5X coverage, supported by paired-end information (where pairs of reads map to locations >300 bp apart that span across the gap region). If detected, deletions were confirmed by contig-building across the gap.

Polymorphic sites were compared across multiple mutants and the sequence of the parent strain. Sites that were heterogeneous (where multiple bases were observed and the majority base was observed in <70% of the reads) or sites in repetitive regions of the genome were screened out, along with differences that appeared across all mutants regardless of resistance, possibly reflecting inaccuracies in the genome sequence of the parental strain. Sites in PE_PGRS genes were also filtered out, as coverage in many of these GC-rich genes is often low, and these potentially polymorphic genes are not essential and not believed to be relevant to mechanisms of drug resistance.

Positions of insertion of the IS*6110* transposon were determined by identifying pairs of reads where one read matches the consensus 1353 bp IS*6110* sequence and the other end maps into the genome. These read pairs were clustered, and the precise insertion coordinates were determined by contig-building, which were then compared to the 16 known insertion locations in the standard H37Rv reference genome.

### Recombineering

Phage Che9 RecT-promoted oligo-mediated recombineering was used to verify the SNPs found in the sequencing of MTb resistant strains. Rolling cultures of *Mtb* H37Rv carrying pNIT::ET (GenBank no. GU459073) [69] were grown at 37°C in 7H9 broth + 30 mg/ml kanamycin to an OD_600_ = 0.5–0.7. Isovaleronitrile was added to a final concentration of 1 mM to induce the recombinase and the cultures were incubated for eight hours at 37°C. Glycine was then added to a final concentration of 1.5% and the cultures were further incubated overnight at 37°C. Competent cells were prepared and electroporations were performed as previously described [10] using 500 ng of a 69 bp DNA oligomer to introduce the SNP. In all cases, a no-DNA control was included and handled in an identical fashion. Following electroporation, the cell mixtures were resuspended in 7H9 (up to 1 ml) and allowed to recover standing overnight at 37°C. Cultures transformed with an oligo, as well as no-DNA controls, were then plated on 7H10 agar + OADC containing inhibitory compounds at concentrations 5 and/or 20-fold higher than the reported MIC, and incubated at 37°C for 3-4 weeks. Verification of the SNP was determined by the presence of resistant colonies on transformation plates spread with cultures transformed with an oligo, and the absence (or near absence) of colonies spread with no-DNA control cultures. The oligo-mediated rate of resistance was between 5-100 fold higher (depending on the SNP) relative to the spontaneous rate (i.e., the no-DNA control). Oligo-dependent resistant colonies were then grown up, the presence of the SNP was verified by sequencing, and MIC’s were determined and compared to wild type *Mtb*. This procedure was used for verifications of SNPs in *mmpL3, pks13, ndhA and aspS*.

A variation of the protocol described above was used for verification of SNPs in *eccB3*, *rv1685c*, *rv0678*, and *ethA*. In this procedure, *Mtb* cells containing plasmid pKM402, which expressed the Che9 RecT recombinase under control of the Ptet promoter, were used for recombineering. This plasmid promotes recombineering frequencies between 10^-3^ to 10^-4^, orders of magnitudes higher than spontaneous mutation frequencies (~10^-8^ to 10^-9^). Thus, resistance due to SNP incorporation can be easily distinguished from spontaneously-induced mutations. The cells were grown as described above, but were induced with 500 nM anhydrotetracycline (Atc) and electroporated with 1 µg of the SNP-encoding oligo. In addition, the cells were co-electroporated with 1 µg of an oligo conferring resistance to streptomycin (via alteration of the *rpsL* gene). Following outgrowth in 5 ml of 7H9 media for 4-5 days, the cells were subcultured into 5 ml of 7H10 containing 20 µg/ml sterptomycin and allowed to grow for an additional 5-7 days. The growth in streptomycin selects for cells that take up DNA, and allows for enrichment of the unselected (SNP-containing) oligo. In trial experiments, 5% of cells that become streptomycin resistance also incorporate the unselected oilgo.

The cultures were then diluted to an Abs_600_ reading for 0.1, and 25 µl of cells (~10^6^) were spotted into 12 wells of a 24 well plate containing 1.5 ml of 7H10 agar containing various concentrations of the inhibitory compound. The concentration range of the compound examined spanned the reported MIC for *Mtb* H37Rv (from 4-fold lower to 20-fold higher). An oligo-dependent increase in the observed MIC of the transformation mixture, relative to a control culture with no DNA added, was used to validate the SNP as the resistant determinant. As further verification, oligo-transformed cells appearing in wells at concentrations above its observed MIC were grown up in 7H9, the target regions were amplified by PCR, and the presence of the SNP was verified by sequencing.

## Supporting Information

File S1 Table S1, Sources of compounds investigated in this study. Table S2, Sequencing details including read length (all were paired-end reads), parental strain, depth of coverage (average number of reads covering each site), completion (percent of sites covered by at least 2 reads), and list of all confident polymorphisms observed for each resistant mutant sequenced. Table S3, qRT PCR studies show that MmpL5 and MmpS5 are significantly up-regulated in the I67S mutant of Rv0678. For comparison, expression levels of MmpL5 and MmpS5 are also shown in two resistant mutants to econazole, suggesting they share the same mechanism of resistance.
(DOC)
