# Supplementary material for: Identification of New Drug Targets and Resistance Mechanisms in Mycobacterium tuberculosis
Source: PLoS One. 2013 Sep 23;8(9):e75245. doi: 10.1371/journal.pone.0075245 (PMC3781026; doi:10.1371/journal.pone.0075245)
Supplement: File S1 — Table S1, Sources of compounds investigated in this study. Table S2, Sequencing details including read length (all were paired-end reads), parental strain, depth of coverage (average number of reads covering each site), completion (percent of sites covered by at least 2 reads), and list of all confident polymorphisms observed for each resistant mutant sequenced. Table S3, qRT PCR studies show that MmpL5 and MmpS5 are significantly up-regulated in the I67S mutant of Rv0678. For comparison, expression levels of MmpL5 and MmpS5 are also shown in two resistant mutants to econazole, suggesting they share the same mechanism of resistance. (DOC) [file pone.0075245.s001.doc]

**Identification of New Drug Targets and Resistance Mechanisms in *Mycobacterium tuberculosis***

Ioerger, T.R. et al.

**Table S1.** Sources of compounds investigated in this study.

| **compound** | **IMTB #** | **PubChem CID** | **Commercial source** | **other source** |
| --- | --- | --- | --- | --- |
| **1** | 034 |  |  | obtained from Dr. Takushi Kaneko, TB Global Alliance |
| **2** | 027 | 5764612 | Asinex BAS 01841799 |  |
| **3** | 028 | 2129850 | Chembridge 6238794 |  |
| **4** | 029 |  | Chembridge 7109595 |  |
| **5** | 036 |  |  | obtained from Dr. Takushi Kaneko, TB Global Alliance |
| **6** | 045 |  |  | obtained from Dr. Takushi Kaneko, TB Global Alliance |
| **7** | 039 | 1037178 | Chembridge 7422868 | Asinex BAS 04370630 |
| **8** | 026 | 2792221 | Chembridge 7497419 |  |

**Table S2.**

Sequencing details including read length (all were paired-end reads), parental strain, depth of coverage (average number of reads covering each site), completion (percent of sites covered by at least 2 reads), and list of all confident polymorphisms observed for each resistant mutant sequenced.

| **compound** | **strain id** | **read length** | **ref strain** | **coverage** | **completion** | **polymorphisms** |
| --- | --- | --- | --- | --- | --- | --- |
| **1** | IMTB034-RM1 | 51x51 bp | H37RvLP | 59.4x | 99.19% | Rv0283:-AAC in aa 26 |
| **1** | IMTB034-RM2 | 47x47 bp | H37RvLP | 139.7x | 99.52% | Rv0283:N24H |
| **1** | IMTB034-RM3 | 47x47 bp | H37RvLP | 163.1x | 99.65% | Rv0283:R14L |
|  |  |  |  |  |  |  |
| **2** | Reiling 2-2 | 51x51 bp | H37RvSB | 26.8x | 98.13% | mmpL3: F644L (g>t), pks5:L907L (a>g) |
| **2** | Reiling 2-4 | 51x51 bp | H37RvSB | 25.7x | 98.24% | mmpL3: F644C (a>c) |
| **2** | Reiling 2-5 | 51x51 bp | H37RvSB | 29.8x | 98.37% | mmpL3: A677V (g>a) |
| **2** | Reiling 2-6 | 51x51 bp | H37RvSB | 21.1x | 97.86% | mmpL3: F644L (g>t), clpX:A86V (g>a), hemY:V298G (a>c), ppsA:W1294* (g>a) |
|  |  |  |  |  |  |  |
| **3** | SBRI 3-1 | 51x51 bp | H37RvSB | 153.8x | 99.34% | pks13 D1644G (t>c) |
| **3** | SBRI 3-3 | 51x51 bp | H37RvSB | 151.1x | 99.11% | pks13 D1607N (c>t), Rv1230 (C360G) (a>c) |
| **3** | SBRI 3-7 | 51x51 bp | H37RvSB | 165.5x | 99.15% | pks13 D1607N (c>t), Rv1230 (C360G) (a>c) |
| **3** | SBRI 3-8 | 51x51 bp | H37RvSB | 165.1x | 99.43% | pks13 D1607N (c>t), Rv1230 (C360G) (a>c) |
|  |  |  |  |  |  |  |
| **4** | SBRI 4-1 | 51x51 bp | H37RvSB | 164.0x | 99.36% | aspS:F526L (g>t) |
| **4** | SBRI 4-2 | 51x51 bp | H37RvSB | 173.9x | 99.40% | aspS:T570I (g>a) |
| **4** | SBRI 4-7 | 51x51 bp | H37RvSB | 165.0x | 99.58% | aspS:T570I (g>a) |
|  |  |  |  |  |  |  |
| **5** | IMTB036-3 | 47x47 bp | H37RvLP | 111.3x | 99.65% | Rv0678:I67S (t>g), pks7:A1034A (c>t) |
| **5** | IMTB036-5 | 47x47 bp | H37RvLP | 154.5x | 99.79% | Rv0678: +GC in aa 69 |
| **5** | IMTB036-6 | 47x47 bp | H37RvLP | 130.9x | 99.52% | Rv0678: -9 +A; mutT1:E252D (g>c); sugI:Q6* (c>t),sugI:T7P (a>c); 3793645:T>C |
|  |  |  |  |  |  |  |
| **6** | IMTB045-1 | 51x51 bp | H37RvSB | 118.5x | 99.62% | IS6110 in Rv1685c at 1911094, NC:4054144:g>t |
| **6** | IMTB045-3 | 51x51 bp | H37RvSB | 120.5x | 99.54% | IS6110 in Rv1685c at 1911121, Rv3267:P209A |
| **6** | IMTB045-4 | 51x51 bp | H37RvSB | 115.8x | 99.73% | IS6110 in Rv1685c at 1911105 |
|  |  |  |  |  |  |  |
| **7** | IMTB039-2 | 47x47 bp | H37RvSB | 126.8x | 99.58% | ethA:C253R (a>g) |
| **7** | IMTB039-RM1 | 51x51 bp | H37RvSB | 121.5x | 99.72% | ethA:-T in aa 24 |
| **7** | IMTB039-RM3 | 51x51 bp | H37RvSB | 87.7x | 99.51% | ethA:-T in aa 113 |
|  |  |  |  |  |  |  |
| **8** | Reiling 1-1 | 51x51 bp | H37RvSB | 27.8x | 97.95% | 472683:G>C -44bp upstream of ndhA |
| **8** | Reiling 1-5 | 51x51 bp | H37RvSB | 28.7x | 98.17% | 472683:G>C -44bp upstream of ndhA |
| **8** | Reiling 1-7 | 51x51 bp | H37RvSB | 26.1x | 97.87% | 472683:G>C -44bp upstream of ndhA |
| **8** | Reiling 1-8 | 51x51 bp | H37RvSB | 26.0x | 98.09% | 472683:G>C -44bp upstream of ndhA |

**Table S3.** qRT PCR studies show that MmpL5 and MmpS5 are significantly up-regulated in the I67S mutant of Rv0678. For comparison, expression levels of MmpL5 and MmpS5 are also shown in two resistant mutants to econazole, suggesting they share the same mechanism of resistance.

|  | **Fold change in expression** | |
| --- | --- | --- |
| **Strain** | **Rv0676c/MmpL5** | **Rv0677c/MmpS5** |
| H37Rv London Pride | 1.0 | 1.0 |
| **5**-resistant mutant (I67S) | 34.3 | 40.4 |
| econazole-resistant mutant 1 | 47.0 | 78.4 |
| econazole-resistant mutant 2 | 48.9 | 51.2 |
